# A systematic scoping review of medicine availability and affordability in Africa

**DOI:** 10.1186/s12913-023-10494-8

**Published:** 2024-01-17

**Authors:** Jeff Lane, Hilma Nakambale, Asha Kadakia, Yoswa Dambisya, Andy Stergachis, Walter Denis Odoch

**Affiliations:** 1 Department of Global Health, School of Public Health, University of Washington, Seattle, WA USA; 2 East Central and Southern Africa Health Community, Arusha, Tanzania; 3 Departments of Pharmacy and Global Health, Schools of Pharmacy and Public Health, University of Washington, Seattle, WA USA; 4 Afya Research and Development Institute, Kampala, Uganda; 5 World Health Organization, Harare, Zimbabwe

**Keywords:** Medicines, Pharmaceuticals, Availability, Affordability, Africa

## Abstract

**Background:**

The most recent World Medicines Situation Report published in 2011 found substantial medicine availability and affordability challenges across WHO regions, including Africa. Since publication of the 2011 report, medicine availability and affordability has risen on the international agenda and was included in the Sustainable Development Goals as Target 3.8. While numerous medicine availability and affordability studies have been conducted in Africa since the last World Medicines Situation Report, there has not been a systematic analysis of the methods used in these studies, measures of medicine availability and affordability, categories of medicines studied, or geographic distribution. Filling this knowledge gap can help inform future medicine availability and affordability studies, design systems to monitor progress toward Sustainable Development Goal Target 3.8 in Africa and beyond, and inform policy and program decisions to improve medicine availability and affordability.

**Methods:**

We conducted a systematic scoping review of studies assessing medicine availability or affordability conducted in the WHO Africa region published from 2009–2021.

**Results:**

Two hundred forty one articles met our eligibility criteria. 88% of the articles (213/241) reported descriptive studies, while 12% (28/241) reported interventional studies. Of the 198 studies measuring medicine availability, the most commonly used measure of medicine availability was whether a medicine was in stock on the date of a survey (124/198, 63%). We also identified multiple other availability methods and measures, including retrospective stock record reviews and self-reported medicine availability surveys. Of the 59 articles that included affordability measures, 32 (54%) compared the price of the medicine to the daily wage of the lowest paid government worker. Other affordability measures were patient self-reported affordability, capacity to pay measures, and comparing medicines prices with a population-level income standard (such as minimum wage, poverty line, or per capita income). The most commonly studied medicines were antiparasitic and anti-bacterial medicines. We did not identify studies in 22 out of 48 (46%) countries in the WHO Africa Region and more than half of the studies identified were conducted in Ethiopia, Kenya, Tanzania, and/or Uganda.

**Conclusion:**

Our results revealed a wide range of medicine availability and affordability assessment methodologies and measures, including cross-sectional facility surveys, population surveys, and retrospective data analyses. Our review also indicated a need for greater focus on medicines for certain non-communicable diseases, greater geographic diversity of studies, and the need for more intervention studies to identify approaches to improve access to medicines in the region.

**Supplementary Information:**

The online version contains supplementary material available at 10.1186/s12913-023-10494-8.

## Background

Lack of access to medicines due to their poor availability or affordability negatively affects health service quality, equity and health outcomes in Africa in a variety of ways. Medicine affordability or availability in Africa is associated with medication adherence [1–3], prescribing decisions [4–6], patient choice of health facility [7–9], patient satisfaction [10–12], care seeking behavior [13, 14], referral patterns [15], compliance with treatment guidelines [16], and health outcomes [1, 17, 18]. Unaffordability of medicines in the formal sector has also been shown to drive patients to informal markets where medicine quality may be poor [19].

The terms medicine availability and medicine affordability do not have universally recognized definitions [20]. As illustrated by the articles identified through this analysis, a range of definitions and measures have been used in the literature to describe the concepts of medicine availability and affordability. For the purposes of this study, we use the term *medicine availability* to mean the degree to which a medicine is physically present at a distribution point (e.g., pharmacy). We use the term *medicine affordability* to refer to the extent to which a medicine can be purchased without causing financial hardship.

In 2011, the World Health Organization (WHO) published the World Medicines Situation Report that included results of nine years of surveys from multiple WHO regions measuring affordability and availability of a basket of essential medicines using the WHO/Health Action International (HAI) cross-sectional facility survey methodology that was originally published in 2003 [21]. An analysis of similar survey results was published by Cameron et al. in 2009 [22]. Prior versions of the World Medicines Situation Report were published in 2004 [23] and 1988 [24]. The 2011 report included the results of medicine availability and affordability surveys from 11 African countries conducted 2001–2008. The surveys from the Africa region reported substantial challenges and variability in the availability and affordability of essential medicines in the public and private sectors. A World Medicines Situation Report has not been published since 2011.

In 2015 the United Nations General Assembly adopted the Sustainable Development Goals (SDGs), which included ensuring “access to safe, effective, quality and affordable essential medicines and vaccines for all” as part of Target 3.8. Following the SDGs declaration, access to medicines has become central to discussions around achievement of Target 3.8. There is a need to better understand the state of medicine availability and accessibility studies in the Africa region since the 2011 Medicines Situation Report to help guide progress toward Target 3.8.

Since the publication of the 2011 Medicines Situation Report, medicine availability and affordability studies using varying methodologies continued to be conducted in the WHO Africa region [25–29]. A range of other methodologies have also been used to measure medicine availability and affordability in Africa, including use of longitudinal medicine stock datasets, in-depth key informant interviews, retrospective stock record reviews, and patient and health worker surveys [30–33]. A small number of systematic reviews have been conducted examining medicine availability and/or affordability in or including Africa with a focus on particular medicines or scenarios (e.g., asthma and chronic obstructive pulmonary disease [34], subsidizing artemisinin-based combination therapy [35], COVID-19 [36], and medicine stock level monitoring using mobile devices [37]). To date, however, there has not been a scoping review to collect and describe the types of medicine availability and affordability studies conducted in the African region across all medicine categories, the methods used in these studies, how medicine availability and affordability are measured, the frequency of different categories of medicines being measured, or the geographic distribution of these studies. A greater understanding of these approaches could provide important context for research on medicine availability and affordability, monitoring progress against SDG Target 3.8, and informing policy and programmatic decision-making to improve medicine availability and affordability in low and middle-income countries.

To address this gap, we conducted a systematic scoping of the literature for medicine availability and affordability surveys and related studies conducted in the WHO Africa Region (48 countries) [38] from 2009 (the year after data collection ended for the medicines availability and affordability surveys included in the 2011 World Medicines Situation Report) through 2021. Systematic scoping reviews are used to identify the types of available evidence in a given field; clarify key concepts and definitions in the literature; examine how research is conducted on a certain topic or field; identify key characteristics or factors related to a concept; and identify and analyze knowledge gaps [39]. The results of this study will help to inform future medicine availability and affordability studies and help design systems to monitor progress toward Sustainable Development Goal Target 3.8 in Africa and beyond. In addition, our findings can inform decision-making for improving medicine availability and affordability by policymakers.

## Methods

We followed the Preferred Reporting Items for Systematic Reviews and Meta-Analyses for Scoping Reviews (PRISMA-ScR) guidelines [40]. The protocol was registered on Open Science Framework on August 11, 2021 (osf.io/t2gdq) [41].

Our inclusion criteria were: (1) analysis of medicines availability or affordability; (2) qualitative or quantitative analysis; (3) geographic focus on countries in WHO Africa Region; (4) English language; (5) published between January 1, 2009 and August 2, 2021 (date of searches). Our exclusion criteria were: (1) not available in English (due to language limitations of study team); (2) assessments of availability/affordability of other types of health supplies/equipment/diagnostics only; (3) last year of data collection was 2005 or earlier.

We conducted searches on August 2, 2021, in the following databases: (1) Medline (PubMed), (2) EMBASE, and (3) WHO Global Index Medicus. The search terms for Medline (PubMed), EMBASE, and WHO Global Index Medicus are listed in Table 1. We reviewed all studies in the HAI Medicine Prices, Availability, Affordability & Price Components Database (https://haiweb.org/what-we-do/price-availability-affordability/price-availability-data/) against our criteria after completing the literature screening and included reports from the HAI database that were not included in the literature results. We de-duplicated the articles using EndNote and Rayyan.

**Table 1:** Search terms

**Database**	**PubMed (Medline)**
Search Terms	((Algeria OR Angola OR Benin OR Botswana OR “Burkina Faso” OR Burundi OR Cabo Verde OR Cameroon OR “Central African Republic” OR Chad OR Comoros OR Congo OR “Cote d’Ivoire” OR “Democratic Republic of Congo” OR “Equatorial Guinea” OR Eritrea OR Eswatini OR Ethiopia OR Gabon OR Gambia OR Ghana OR “Guinea Bissau” OR Kenya OR Lesotho OR Liberia OR Madagascar OR Malawi OR Mali OR Mauritania OR Mauritius OR Mozambique OR Namibia OR Niger OR Nigeria OR Rwanda OR “Sao Tome and Principe” OR Senegal OR Seychelles OR “Sierra Leone” OR “South Africa” OR “South Sudan” OR Togo OR Uganda OR Tanzania OR Zambia OR Zimbabwe) AND (“medicine*”[tiab] OR “pharmaceutical*” OR “Pharmaceutical Preparations” [Mesh]) AND (“affordability” or “availability”)) AND ((“2009”[Date—Publication]: “3000”[Date—Publication]))
Date Search Conducted	2 Aug 2021
Number of Results	1126
**Database**	**Embase**
Search Terms	((‘algeria’ OR ‘algeria’/exp OR algeria OR ‘angola’ OR ‘angola’/exp OR angola OR ‘benin’ OR ‘benin’/exp OR benin OR ‘botswana’ OR ‘botswana’/exp OR botswana OR ‘burkina faso’/exp OR ‘burkina faso’ OR ‘burundi’ OR ‘burundi’/exp OR burundi OR cabo) AND verde OR ‘cameroon’ OR ‘cameroon’/exp OR cameroon OR ‘central african republic’/exp OR ‘central african republic’ OR ‘chad’ OR ‘chad’/exp OR chad OR ‘comoros’ OR ‘comoros’/exp OR comoros OR ‘congo’ OR ‘congo’/exp OR congo OR ‘cote divoire’ OR ‘democratic republic of congo’ OR ‘equatorial guinea’/exp OR ‘equatorial guinea’ OR ‘eritrea’ OR ‘eritrea’/exp OR eritrea OR ‘eswatini’ OR ‘eswatini’/exp OR eswatini OR ‘ethiopia’ OR ‘ethiopia’/exp OR ethiopia OR ‘gabon’ OR ‘gabon’/exp OR gabon OR ‘gambia’ OR ‘gambia’/exp OR gambia OR ‘ghana’ OR ‘ghana’/exp OR ghana OR ‘guinea bissau’/exp OR ‘guinea bissau’ OR ‘kenya’ OR ‘kenya’/exp OR kenya OR ‘lesotho’ OR ‘lesotho’/exp OR lesotho OR ‘liberia’ OR ‘liberia’/exp OR liberia OR ‘madagascar’ OR ‘madagascar’/exp OR madagascar OR ‘malawi’ OR ‘malawi’/exp OR malawi OR ‘mali’ OR ‘mali’/exp OR mali OR ‘mauritania’ OR ‘mauritania’/exp OR mauritania OR ‘mauritius’ OR ‘mauritius’/exp OR mauritius OR ‘mozambique’ OR ‘mozambique’/exp OR mozambique OR ‘namibia’ OR ‘namibia’/exp OR namibia OR ‘niger’ OR ‘niger’/exp OR niger OR ‘nigeria’ OR ‘nigeria’/exp OR nigeria OR ‘rwanda’ OR ‘rwanda’/exp OR rwanda OR ‘sao tome and principe’/exp OR ‘sao tome and principe’ OR ‘senegal’ OR ‘senegal’/exp OR senegal OR ‘seychelles’ OR ‘seychelles’/exp OR seychelles OR ‘sierra leone’/exp OR ‘sierra leone’ OR ‘south africa’/exp OR ‘south africa’ OR ‘south sudan’/exp OR ‘south sudan’ OR ‘togo’ OR ‘togo’/exp OR togo OR ‘uganda’ OR ‘uganda’/exp OR uganda OR ‘tanzania’ OR ‘tanzania’/exp OR tanzania OR ‘zambia’ OR ‘zambia’/exp OR zambia OR ‘zimbabwe’ OR ‘zimbabwe’/exp OR zimbabwe) AND (‘medicine*’:ti,ab,kw OR ‘pharmaceutical*’:ti,ab,kw OR ‘prescription drug’/exp) AND (‘affordability’/exp OR ‘affordability’ OR ‘availability’/exp OR ‘availability’) AND [2009–2021]/py AND [english]/lim
Date Search Conducted	2 Aug 2021
Number of Results	1,220
**Database**	**WHO Global Index Medicus**
Search Terms	((mh:(vs2.002.001*)) OR medicine* OR pharmaceutical*) AND (“affordability” OR “availability”) AND ((mh:( Z01.058*)) OR (collection_gim:("AIM")))
Date Search Conducted	2 Aug 2021
Number of Results	135

*denotes wildcard search symbol

Three authors (JL, HN, and AK) independently screened each identified record by title and abstract based on the above inclusion and exclusion criteria using Rayyan [42]. JL independently reviewed all records and HN and AK each reviewed 50% of the abstracts. Any disagreements were resolved via discussion by the review authors. Three authors (JL, HN, and AK) then independently reviewed the full text of screened articles based on the above inclusion and exclusion criteria and entered review decision in Rayyan. JL reviewed all screened full text articles and AK and HS each reviewed 50% of the screened full text articles. Any disagreements were resolved via discussion by the three review authors.

Data extraction was conducted by JL. The following data elements were extracted into Excel and descriptive statistics were used to analyze the extracted data: Study ID; authors; article title; publication year; data collection year(s); journal title; country(ies) where study conducted; type of study (free text); type of study (select from descriptive, correlation, or intervention); types of medicines studied; types of facilities/locations studied; types of respondents (if applicable); availability measure(s); and affordability measure(s). The dataset is available as a Supplemental File 1.

Types of medicines were categorized according to the categories of medicines used in the 2021 WHO Model List of Essential Medicines (WHO Model EML). The highest level category was used for all categories, except anti-infective medicines, for which we also used the sub-categories due to the large number of medicines in the sub-categories (i.e., anthelminthics, antibacterials, antifungal medicines, antiviral medicines, antiprotozoal medicines, and medicines for ectoparasitic infections). The categories are listed in Table 2. Some medicines are coded to multiple categories.

**Table 2:** Coding scheme for type(s) of medicines studied

No. from 2021 WHO Model EML	Category Name
1	Anaesthetics, Preoperative Medicines and Medical Gases
2	Medicines for Pain and Palliative Care
3	Antiallergics and Medicines Used in Anaphylaxis
4	Antidotes and Other Substances Used in Poisonings
5	Anticonvulsants/Antiepileptics
6	Anti-Infective Medicines
6.1	Anthelminthics
6.2	Antibacterials
6.3	Antifungal medicines
6.4	Antiviral medicines
6.5	Antiprotozoal medicines
6.6	Medicines for ectoparasitic infections
7	Antimigraine Medicines
8	Immunomodulators and Antineoplastics
9	Antiparkinsonism Medicines
10	Medicines Affecting the Blood
11	Blood Products of Human Origin and Plasma Substitutes
12	Cardiovascular Medicines
13	Dermatological Medicines (topical)
14	Diagnostic Agents
15	Antiseptics and Disinfectants
16	Diuretics
17	Gastrointestinal Medicines
18	Medicines for Endocrine Disorders
19	Immunologicals
20	Muscle Relaxants (peripherally-acting) and Cholinesterase Inhibitors
21	Ophthalmological Preparations
22	Medicines for Reproductive Health and Perinatal Care
23	Peritoneal Dialysis Solution
24	Medicines for Mental and Behavioural Disorders
25	Medicines Acting on the Respiratory Tract
26	Solutions Correcting Water, Electrolyte and Acid–Base Disturbances
27	Vitamins and Minerals
28	Ear, Nose and Throat Medicines
29	Medicines for Diseases of Joints
30	Dental Preparations
N/A^a^	Not specific to a particular medicine

^a^We coded some studies as “Not specific to a particular medicine” which was not a category in the WHO Model EML

The categorization decisions were based on a review of three resources. First, we reviewed the 2021 WHO Model EML. Medicines listed on the 2021 WHO Model EML were coded based on the category assigned in that list. If a medicine was not listed on the 2021 WHO Model EML, we reviewed the online WHO EML website to determine if the medicine had been listed on a prior WHO EML. If so, we categorized the medicine based on category(ies) assigned in the previous WHO Model EML. If the medicine had not been listed on any previous WHO Model EML, we reviewed the MedLine Plus National Library of Medicine to inform the categorization decision. The coding decisions for medicines not listed on the 2021 WHO Model EML are listed in the coding notes of Supplemental File 1 under the WHO EML Categories tab.

## Results

**Fig. 1:**
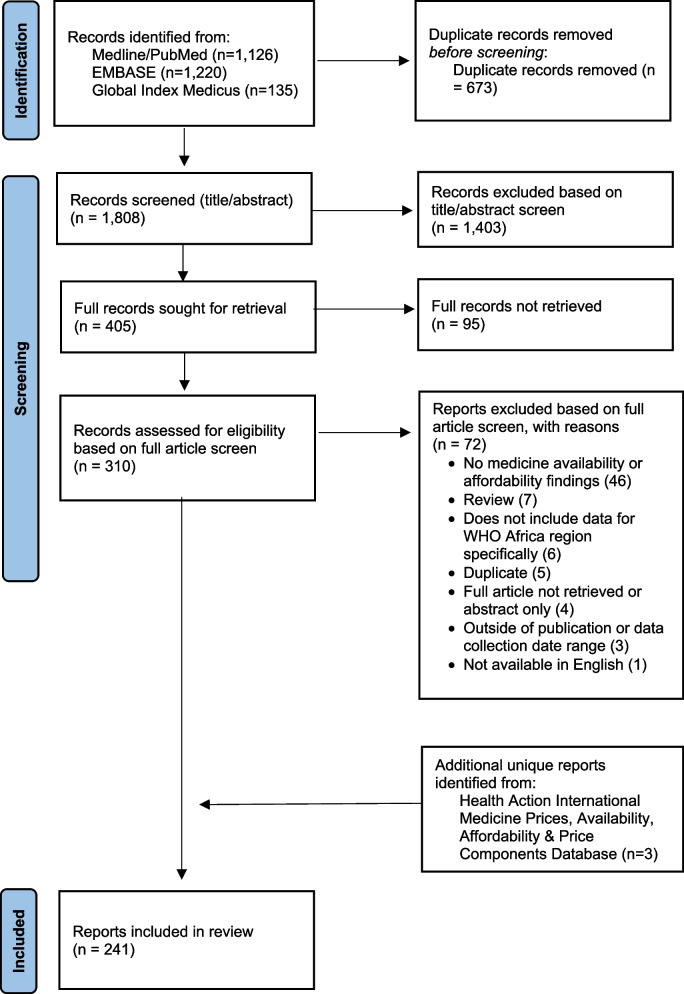
PRISMA Flow diagram of record inclusion/exclusion decisions

Our searches of Medline/PubMed, EMBASE, and Global Index Medicus identified 2,481 total results (1,126 from Medline/PubMed, 1,220 from EMBASE, and 135 from Global Index Medicus). We used EndNote and Rayyan to de-duplicate the search results, resulting in 1,808 articles for abstract screening. Based on our review of article abstracts, we identified 405 abstracts as potentially meeting our criteria and were able to retrieve 310 articles for full text screening. We excluded 72 articles during the full text screening resulting in 238 articles in our data. We identified three (3) additional reports in the HAI Medicine Prices, Availability, Affordability & Price Components Database that were not duplicates of already included studies, resulting in a total of 241 records in our final dataset. The full list of records included in the analysis, including title, authors, journal title, and year published, data collection year, location, type of study, availability measures, affordability measures, and WHO EML categories, is included in Supplemental File 1. The tabs in Supplemental File 1 allows the reader to sort and identify articles using these characteristics. The list of articles excluded at the full text screening stage and reasons for exclusion are listed in Supplemental File 2. The outcomes of the inclusion and exclusion process are shown in the PRISMA flow diagram (Fig. 1). A PRISMA-ScR checklist is included as Supplemental File 3.

### Types of studies

Most articles (213/241; 88%) presented descriptive studies. Only 12% (28/241) of all the articles were intervention studies that examined the potential effect of programmatic or other interventions on medicine availability and/or affordability. Of the descriptive studies, 27% (57/213) used statistical analytical techniques to identify associations between medicine availability and/or affordability measures and other variables, such as medication adherence [2], use of medicines [43], or compliance with treatment guidelines [16]. The design of each study can be identified in Supplemental File 1 using the Type of Study tab.

### Availability measures

We identified 198 articles (198/241, 82%) that included studies applying a medicine availability measure. Of the articles applying an availability measure, the most common measure was whether a medicine was in stock on the date of a survey (i.e., cross-sectional survey) (124/198, 63%). This included, but was not limited to, studies that used the WHO/HAI survey methodology. The second most common measure was whether stockouts occurred during a particular time period (48/198, 24%). Often these two measures were combined. For example, Iwu et al. combined the first two measures to assess the occurrence of stockouts of six tracer vaccines in Eastern Cape, South Africa [44]. They assessed whether the vaccine was available on the date of a survey and in the preceding 24 months using a questionnaire, record checks, and observation. The third most common medicine availability measure was respondent self-reported availability of a particular medicine (18/198, 9%). Aweucha et al. used this approach to examine the impacts of the COVID-19 pandemic on patient access to essential medicines [45]. They implemented a cross-sectional survey using electronic questionnaires across 36 states of Nigeria by asking patients whether they had “Difficulty accessing essential medicines” before and/or during the COVID-19 pandemic. Six articles (6/198, 3%) used a measure of whether a medicine was on a stock list only. Five articles (5/198, 3%) used a measure of the amount of stock available for a particular medicine. Kusemererwa et al. used a stock level measure to assess medicine availability in the Uganda’s public sector [46]. They measured facility stock levels and characterized stock as optimally stocked, understocked, or overstocked. An item was considered optimally stocked if the facility had two to five months of stock, understocked if it had less than two months, and overstocked if it had more than five months. To calculate the months of stock on hand, the authors divided the stock level on the day of the study by its average monthly consumption. We also identified three articles (3/198, 2%) that used prescription refill data to inform their assessment of medicine availability. Table 3 summarizes the primary availability methods and measures used by the studies in our review. The studies applying each type of availability measure can be identified in Supplemental File 1 using the Availability Measures tab.

**Table 3:** Medicine availability methods & measures

Availability Methods	Example Availability Measures
**Cross-sectional facility stock surveys** (124/198, 63%)^a^	Whether medicine was in stock on a certain date (e.g., using identified surveyor or mystery shopper)
**Retrospective review of facility or system-level medicine stock data/records (e.g., paper bin cards, routine data systems)** (48/198, 24%)	Whether stockout occurred during date range (e.g., 1, 6, or 12 months)
**Patient or health worker self-reported availability** (18/198, 9%)	(Ask) how often certain medicines are available/unavailable based on their experience (e.g., never, rarely, sometimes, most often, always) (Ask) whether they agree with a statement that describes the goal of consistent availability of medicines or the problem of unavailability of medicines (e.g., my facility has enough [insert name of medicine]; I am limited by the unavailability of [insert name of medicine])
**Review of medicine stock lists** (6/198, 3%)	Whether medicine was included on the facility stock list
**Stock level assessment** (5/198, 3%)	Daily stock levels during date range (e.g., stockout, low stock, medium stock, high stock)
**Prescription fill and/or refill data** (3/198, 2%)	Prescription fill or refill rate during date range

^a^Parenthetical indicates the number and percentage of articles using type of method

### Affordability measures

We identified 59 articles (59/241, 24%) presenting studies that included measures of medicine affordability. Of the studies that included an affordability measure, the most common affordability measure compared the price of the medicine to the daily wage of the lowest paid government worker (32/59, 54%). This is the measure used in the WHO/HAI methodology. The second most common affordability measure was patient self-reported affordability (9/59, 15%). The methodology employed by Embrey et al. included a household survey in Tanzania that asked respondents whether they “had to sell things or borrow money to pay for medicines at some time in the past” and whether the household could “usually afford to buy needed medicines” [47]. Oridanigo et al. used both a self-reported/perceived affordability measure and a standardized measure (i.e., daily wages of the lowest paid government worker) to measure medicine affordability in Ethiopia [48]. Oyando et al. conducted a patient survey in Kenya to assess affordability of hypertension care and asked patients if they did any of the following to cover hypertension care costs: “borrowing (having taken a loan), selling household items or assets (eg, livestock), and use of savings” [49]. Five of the articles applying an affordability measure (5/59, 8%) reported studies that used capacity to pay or similar calculation based on individual income and expenses. Khatib et al. [50] and Attaei et al. [1] characterized medicines as affordable if the combined cost was less than 20% of household capacity-to-pay. Capacity-to-pay was calculated based on the household income remaining after basic subsistence needs have been met. A smaller number of studies measured affordability by comparing medicines prices with a population-level income standard such as per capita income (4/59, 7%), minimum wage (4/59, 7%), or the national poverty line (1/59, 2%). For example, Khuluza and Haefele-Abah used the statutory minimum daily wage of Malawi as the affordability threshold [51]. Table 4 summarizes the primary affordability methods and measures used by the studies in our review. The studies applying each type of affordability measure can be identified in Supplemental File 1 using the Affordability Measures tab.

**Table 4:** Medicine affordability methods & measures

Affordability Methods	Affordability Measures
**Compare medicine prices with population-level affordability standard** (41/59, 69%)^a^	Daily wage of lowest paid government worker
	Daily per capita income
	Daily minimum wage
	National poverty line
**Calculate percentage of actual household income spent on medicines** (5/59, 8%)	Percent of actual household income spent on medicines
	Capacity-to-pay measures (e.g., % of household income spent on medicines after covering basic subsistence needs)
**Self-reported affordability of medicines** (9/59, 15%)	Ask whether the household could usually afford to buy needed medicines
	Ask whether they took any steps that might indicate financial hardship to buy medicines (e.g., borrowing money, taking out a loan, selling household items or assets, or using savings intended for another purpose)

^a^Parenthetical indicates the number and percentage of articles using type of method or measure

### Types of medicines studied

Table 5 shows the number of articles that analyzed medicine availability and/or affordability for different categories of medicines. The categories of medicines most commonly studied were antiprotozoal medicines (100/241, 41%) (primarily antimalarials) and antibacterials (93/241, 39%), followed by cardiovascular medicines (70/241, 29%), gastrointestinal medicines (66/241, 27%), medicines for reproductive health and perinatal care (65/241, 27%), medicines for pain and palliative care (62/241, 26%), and medicines for endocrine disorders (62/241, 26%). We did not identify any articles on medicines for ectoparasitic infections, peritoneal dialysis solution, or dental preparations.

**Table 5:** Types of medicines studied

Medicine Category	No. of Articles (%)	Medicine Category (continued)	No. of Articles (%)
Antiprotozoal medicines	100 (41%)	Diuretics	23 (10%)
Antibacterials	93 (39%)	Anaesthetics, Preoperative Medicines and Medical Gases	21 (9%)
Cardiovascular Medicines	70 (29%)	Dermatological Medicines (topical)	21 (9%)
Gastrointestinal Medicines	66 (27%)	Anthelminthics	20 (9%)
Medicines for Reproductive Health and Perinatal Care	65 (27%)	Vitamins and Minerals	20 (8%)
Medicines for Pain and Palliative Care	62 (26%)	Immunologicals	17 (7%)
Medicines for Endocrine Disorders	62 (26%)	Antifungal medicines	15 (6%)
Medicines Acting on the Respiratory Tract	46 (19%)	Medicines for Diseases of Joints	14 (6%)
Antimigraine Medicines	45 (19%)	Ear, Nose and Throat Medicines	7 (3%)
Anticonvulsants/Antiepileptics	43 (18%)	Antiseptics and Disinfectants	6 (2%)
Ophthalmological Preparations	37 (15%)	Antidotes and other Substances used in Poisonings	5 (2%)
Medicines for Mental and Behavioural Disorders	37 (15%)	Blood Products of Human Origin and Plasma Substitutes	4 (2%)
Not specific to a particular medicine	35 (15%)	Antiparkinsonism Medicines	3 (1%)
Antiviral medicines	28 (12%)	Diagnostic Agents	1 (< 1%)
Antiallergics and Medicines used in Anaphylaxis	27 (11%)	Muscle Relaxants (peripherally-acting) and Cholinesterase Inhibitors	1 (< 1%)
Immunomodulators and Antineoplastics	26 (11%)	Medicines for ectoparasitic infections	0 (0%)
Solutions Correcting Water, Electrolyte and Acid–base Disturbances	25 (10%)	Peritoneal Dialysis Solution	0 (0%)
Medicines Affecting the Blood	24 (10%)	Dental Preparations	0 (0%)

### Study locations

Table 6 shows the study location(s) by country. Our results included studies from 26 out of 48 (54%) countries in the WHO Africa Region. The countries with the greatest number of studies were Tanzania (50), Uganda (49), Ethiopia (35), Kenya (33), Nigeria (29), and Ghana (21). Some articles included study sites in multiple countries. The list of study sites for each article is listed in Supplemental File 1 and can be identified using the Location tab.

**Table 6:** Study location(s) by country

Country	No. of Articles (%)	Country (continued)	No. of Articles (%)
Tanzania	50 (21%)	Senegal	6 (3%)
Uganda	49 (20%)	Benin	5 (2%)
Ethiopia	35 (15%)	Burkina Faso	4 (2%)
Kenya	33 (14%)	Sierra Leonne	4 (2%)
Nigeria	29 (12%)	Madagascar	3 (1%)
Ghana	21 (9%)	Mali	3 (1%)
South Africa	15 (6%)	Zimbabwe	3 (1%)
Zambia	12 (5%)	Botswana	2 (1%)
Malawi	10 (4%)	Gambia	2 (1%)
Cameroon	7 (3%)	Swaziland	2 (1%)
Democratic Republic of Congo	7 (3%)	Burundi	1 (< 1%)
Rwanda	7 (3%)	Congo	1 (< 1%)
Mozambique	6 (3%)	Lesotho	1 (< 1%)

## Discussion

Our review set out to describe the types of medicine availability and affordability studies conducted in the African region since the 2011 World Medicines Situation Report, the methods used in these studies, how medicine availability and affordability are measured, the frequency of different categories of medicines studied, and the geographic distribution of these studies. Our findings build on prior reviews [34–37] by including all medicine categories studied across the WHO Africa Region.

Our finding that 88% of articles presented descriptive studies illustrates the importance of descriptive studies to this topic, but also indicated a potential lack of intervention studies exploring approaches to improve the availability and affordability of medicines in the region. Only 12% of the studies in our review were categorized as intervention studies. Studies examining the effect of specific policy and programmatic interventions on medicine availability and affordability will be important to translate research on medicine availability and affordability into actionable policies and programs that increase access to these medicines.

The extensive use of mixed methods approaches in our results was also noteworthy. Many of the studies in our results included a supply-side medicine availability survey and some type of qualitative methods component, such as key informant interviews, questionnaires, or surveys. The Environmental Profile of a Community’s Health (EPOCH) instrument used by Attaei et al. is a good example of a mixed method approach that used “direct observation of the physical and commercial environment and a survey of perceptions of the environment by those living in it” to assess availability and affordability blood-pressure lowering medicines in 20 countries [1].

The large percentage of studies using cross-sectional study designs (either consistent with or similar to the WHO/HAI methodology), shows the continued importance and impact of the WHO/HAI methodology in encouraging standardized survey designs. This standardization facilitates aggregating results from multiple surveys within and between countries and measuring longitudinal change. HAI has maintained a database of studies that have used this particular methodology that facilitates cross-study comparisons.

We also found a substantial number of studies using alternative approaches to measure medicine availability and/or affordability that may complement the WHO/HAI methodology. For example, 24% of the articles with availability studies used a retrospective review of stock records to capture longitudinal data on medicine availability. These longitudinal studies can measure availability over time and provide additional insight on seasonality or other factors that can affect medicine stock rates, such as whether low stock or stockouts occur more frequently at the end of a month/quarter or during certain times of year (e.g., during the rainy season).

For affordability studies, 54% (32/59) of the articles with affordability studies used the WHO/HAI methodology of comparing medicine prices with the daily wages of the lowest paid government worker. However, 15% (9/59) of the articles with affordability studies used patient self-reported affordability measures and 8% (5/59) used actual household-level income and expense data to assess affordability. Furthermore, 15% (9/59) used a population-level threshold for affordability other than the wages of the lowest paid government worker, such as per capita income, minimum daily wage, or national poverty line.

Many studies in our results collected primary data, but a substantial number of studies relied on data collected through largescale surveys, such as the Service Availability and Readiness Assessment survey [52], or accessed routine data systems with data collected and maintained by a government agency. Integrating medicine availability and affordability questions into population-level survey instruments may present an opportunity to monitor medicine availability and affordability at the population-level at minimal additional costs. We have also seen countries publish medicine availability and distribution data on public dashboards during the COVID-19 pandemic, and these dashboards and their underlying datasets may present opportunities for collaborative medicine availability or affordability monitoring between researchers and government agencies. For example, South Africa established a COVID-19 public dashboard that showed COVID-19 vaccine administration and coverage data [53] and the U.S. established a medicine availability dashboard for COVID-19 therapeutics that showed stock levels at more than 33,000 public and private health facilities [54]. Due to the costs associated with conducting primary data collection, incorporating medicine availability and affordability questions into existing population survey questionnaires and leveraging routine data systems of ministries and departments of health may present opportunities for efficient population-level monitoring of medicine availability and affordability.

The most commonly studied medicines in our results were antiprotozoal medicines, including antimalarials, and antibacterials, including anti-TB medicines. Antiprotozoal medicines being the most studied category of medicines reflects the substantial burden of malaria in Africa and the important role of antimalarial medicines in reducing malaria morbidity and mortality. Antibacterials being the second most commonly studied medicine also illustrates the focus of addressing the burden of infectious diseases in Africa, including the high burden of tuberculosis in many countries in the region. However, medicines for non-communicable diseases, such as cardiovascular, reproductive health and perinatal care, gastrointestinal, and endocrine disorders, were also examined in a large number of studies, signifying the double burden of communicable and non-communicable diseases within the countries. Our results only identified 26 articles that included medicines for immunomodulators and antineoplastics, such as anti-cancer medicines. As the burden of disease in Africa continues to shift toward non-communicable diseases, there will be an increasing need for medicine availability and affordability studies for these types of medicines.

Our review found unequal distribution of medicine availability and affordability studies across the WHO Africa Region. We did not identify any medicine availability or affordability studies in 22 out of 48 (46%) countries in the WHO Africa Region. Moreover, of the 26 countries where studies had occurred, more than half were conducted in the combination of Ethiopia, Kenya, Tanzania, and/or Uganda. Increasing the coverage of medicine availability and affordability studies across the region will be important for monitoring progress toward SDG Target 3.8.

Our review was limited by only being able to review manuscripts published in English (due to language limitations of the authors), which may have contributed to the lack of studies identified from countries where English is not widely used. We also made an adjustment to the original protocol by adding an additional exclusion criteria to exclude articles for which the last year of data collection was 2005 or earlier. This additional inclusion criteria was not originally contemplated in the protocol, but we felt important to maintain the scope to our time period of interest. Nevertheless, our review was able to include a very large number of articles (241), which we believe provides an important and timely review of medicine availability and affordability studies in the WHO Africa Region since publication of the 2011 World Medicines Situation Report.

## Conclusions

Our review revealed a range of methodologies and measures being used to study the availability and/or affordability of medicines across Africa. We identified studies measuring medicine availability using cross-sectional survey design, key informant qualitative interviews, respondent surveys, longitudinal stock record reviews, and pharmacy prescribing data. The results showed the important role that the WHO/HAI methodology has played in standardizing medicine availability and affordability surveys across the region and the emerging role other methodologies are playing in measuring medicine availability and affordability. While the majority of affordability studies used the wages of the lowest paid government worker as a population-level proxy for medicine affordability, we also found a number of other population-level proxy measures (e.g., minimum wage, per capita income, or poverty line). We also found other affordability measured being applied, including calculating percentage of actual household income spent on medicines and/or self-reported affordability of medicines using population surveys or interviews.

As the burden of non-communicable diseases increases in Africa, there will be an increasing need to focus medicine availability and affordability studies on medicines for non-communicable conditions and greater funding and focus may be needed for intervention studies to help identify systems and processes that may increase access to these medicines. Our hope is that this review and the methods and measures described herein will be a useful reference for researchers and governments in designing studies and routine monitoring systems to measure and ultimately improve the availability and affordability of medicines in the region.

### Supplementary Information

**Additional file 1.**

** Additional file 2.**

** Additional file 3.**

## Data Availability

The underlying data file is included with this manuscript as a supplemental files.

## Abbreviations

EML: Essential Medicines List
EPOCH: Environmental Profile of a Community’s Health
HAI: Health Action International
SDG: Sustainable Development Goals
WHO: World Health Organization
